# A phospholipid:diacylglycerol acyltransferase is involved in the regulation of phospholipids homeostasis in oleaginous *Aurantiochytrium* sp.

**DOI:** 10.1186/s13068-023-02396-y

**Published:** 2023-09-27

**Authors:** Huidan Zhang, Zhuojun Wang, Caili Sun, Chuchu Zhang, Huan Liu, Qiu Cui, Xiaojin Song, Sen Wang

**Affiliations:** 1 CAS Key Laboratory of Biofuels, Qingdao Institute of Bioenergy and Bioprocess Technology, Chinese Academy of Sciences, No.189 Songling Road, Laoshan District, Qingdao, 266101 Shandong China; 2 Academy of Plateau Science and Sustainability, Qinghai Normal University, Xining, 810016 Qinghai China; 3 Shandong Provincial Key Laboratory of Energy Genetics, Shandong Engineering Laboratory of Single Cell Oil, Shandong Energy Institute, Qingdao, 266101 Shandong China; 4 Qingdao New Energy Shandong Laboratory, Qingdao Engineering Laboratory of Single Cell Oil, Qingdao, 266101 Shandong China; 5 Key Laboratory of Marine Drugs, Ministry of Education, School of Medicine and Pharmacy, Ocean University of China, Qingdao, 266003 China

**Keywords:** *Aurantiochytrium*, Phospholipid:diacylglycerol acyltransferase, Triacylglycerol, Phospholipids, Cell number

## Abstract

**Background:**

Thraustochytrids have gained attention as a potential source for the production of docosahexaenoic acid (DHA), where DHA is predominantly stored in the form of triacylglycerol (TAG). The TAG biosynthesis pathways, including the acyl-CoA-dependent Kennedy pathway and the acyl-CoA-independent pathway, have been predicted in thraustochytrids, while the specific details regarding their roles are currently uncertain.

**Results:**

Phospholipid:diacylglycerol acyltransferase (PDAT) plays a key role in the acyl-CoA-independent pathway by transferring acyl-group from phospholipids (PL) to diacylglycerol (DAG) to from TAG. In thraustochytrid *Aurantiochytrium* sp. SD116, an active *Au*PDAT was confirmed by heterologous expression in a TAG-deficient yeast strain H1246. Analysis of *Au*PDAT function in vivo revealed that deletion of *Au*PDAT led to slow growth and a significant decrease in cell number, but improved PL content in the single cell during the cell growth and lipid accumulation phases. Interestingly, deletion of *Au*PDAT did not affect total lipid and TAG content, but both were significantly increased within a single cell. Moreover, overexpression of *Au*PDAT also resulted in a decrease in cell number, while the total lipid and cell diameter of a single cell were markedly increased. Altogether, both up-regulation and down-regulation of *Au*PDAT expression affected the cell number, which further associated with the total lipid and TAG content in a single cell.

**Conclusions:**

Our study demonstrates that *Au*PDAT-mediated pathway play a minor role in TAG synthesis, and that the function of *Au*PDAT may be involved in regulating PL homeostasis by converting PL to TAG in a controlled manner. These findings expand our understanding of lipid biosynthesis in *Aurantiochytrium* sp. and open new avenues for developing “customized cell factory” for lipid production.

**Supplementary Information:**

The online version contains supplementary material available at 10.1186/s13068-023-02396-y.

## Background

Microbial oils are gaining attention due to their potential as biofuel or nutraceuticals [1]. Several typical oleaginous microorganisms, such as *Thraustochytrids*, *Mucor circinelloides*, *Mortierella isabellina*, and *Yarrowia lipolytica*, have been well used for commercial lipid production [2]. Lipids in oleaginous microorganisms consist of triacylglycerol (TAG), phospholipids (PL) and glycolipid, in which TAG is the primary component [3, 4]. In eukaryotes, TAG is synthesized through the acyl-CoA-dependent Kennedy pathway or the acyl-CoA-independent pathway [5]. The Kennedy pathway includes several enzymes such as acyl-CoA:glycerol-sn-3-phosphate acyltransferase (GPAT), lysophosphatidate acyltransferase (LPAT), phosphatidic acid phosphatase (PAP), and diacylglycerol acyltransferase (DGAT). DGAT has been proposed to be the rate-limiting enzyme in TAG biosynthesis [6]. Additionally, the acyl-CoA-independent pathway, which is mediated by phospholipid:diacylglycerol acyltransferase (PDAT), can also synthesize TAG [7]. PDAT can transfer an acyl-group from PL to diacylglycerol (DAG) to from TAG. This characteristic has been identified in yeasts [8], algae [7], and plants [9]. In *Saccharomyces cerevisiae*, both PDAT and DGAT are the major contributors to TAG biosynthesis [10]. And heterologous expression of *Cr*PDAT from *Chlamydomonas reinhardtii* could efficiently divert fatty acid flux from PL into TAG in *Escherichia coli* [11]. These suggest that regulating the expression of PDAT may enhance the TAG accumulation in oleaginous microorganisms.

Thraustochytrids are regarded as the promising docosahexaenoic acid (DHA) producers because of their high growth rate, lipid content and DHA content [12]. DHA is beneficial for human health due to its roles in the development of the brain and vision in newborns [13]. In thraustochytrids, both the Kennedy pathway and PDAT-mediated pathway were discovered for TAG synthesis by predicting the encoding genes [3]. Functions of DGATs in Kennedy pathway have been studied in thraustochytrid *Aurantiochytrium* sp. [14, 15], and results showed that overexpression of endogenous DGATs can significantly improve lipid production [15]. Several studies have reported the transferring process of fatty acyl moiety from PC to DAG for TAG biosynthesis in thraustochytrid [3, 16]. Zhao et al. report that unlike other oleaginous microbes, the very long chain polyunsaturated fatty acids, including DHA, accumulated in TAG in Thraustochytrium are channeled from phosphatidylcholine [16]. Meanwhile, Yue et al. suggested that DHA is primarily accumulated in PL and then migrated from PL to TAG during the fermentation cycle [3]. The acyltransferase activity of PDATs has been confirmed in yeast, algae, and plant [7, 17]. This suggests that the migrating process of DHA from PL to TAG may mediate by PDAT in thraustochytrid, but its physiologic role is uncertain.

In this study, we investigated the role of PDAT-mediated TAG biosynthesis pathway in thraustochytrid *Aurantiochytrium* sp. An intact PDAT was predicted in the *Aurantiochytrium* sp. SD116’s genome, and its function was determined by ex vivo and in vivo experiments. Based on the results, the function of PDAT may involve regulating PL homeostasis by properly converting PL to TAG. The investigation of PDAT in *Aurantiochytrium* sp. increases our understanding of lipid biosynthesis and creates new opportunities to develop a “customized cell factory” for lipid production.

## Results

### *Au*PDAT exhibits acyltransferase activity

Using *Hondaea fermentalgiana* PDAT (*Ho*PDAT, GeneBank Accession Number: GBG32689.1) as probe, a putative PDAT (GeneBank Accession Number: OP715731) was identified in *Aurantiochytrium* sp. SD116 via a blast search. It is composed of 783 amino acids with a molecular weight of 87 kDa and exhibits 60% similarity to *Ho*PDAT. SMART (http://smart.embl-heidelberg.de/) predicted that the putative PDAT contains a PGAP1 domain and a lecithin:cholesterol acyltransferase (LCAT) domain. PGAP1 is an endoplasmic reticulum (ER) membrane protein that functions as a glycosylphosphatidylinositol-deacylase [18], and LCAT catalyzes the esterification of DAG using phosphatidylcholine (PC) as the acyl donor [17]. Phylogenetic analysis revealed the putative PDAT is closely related to the PDAT of *H. fermentalgiana* but distantly related to PDATs in plants and most fungus (Additional file 1). Both *H. fermentalgiana* and *Aurantiochytrium* sp. belong to thraustochytrids, while roles of these PDATs in thraustochytrids are still unknown.

**Fig. 1:**
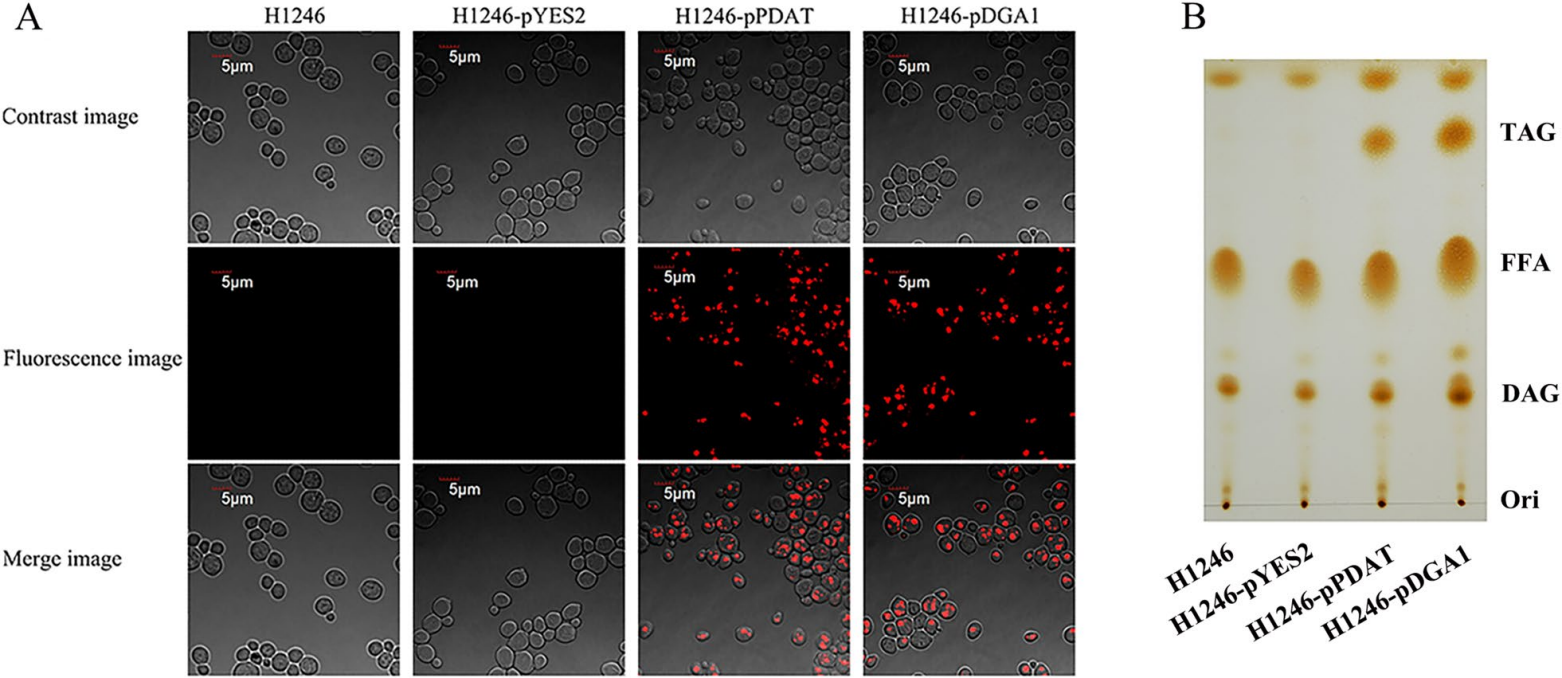
Characterization of acyltransferase activity of *Au*PDAT in TAG-deficient *S. cerevisiae* H1246 cells. **A** Nile red (NR) staining analysis of H1246 cells and its transformants. **B** TLC analysis of lipids from H1246 and its transformants. H1246-pYES2, H1246 harboring the empty plasmid pYES2; H1246-pPDAT, H1246 expressing *Au*PDAT; H1246-pDGA1, H1246 expressing yeast DGA1

Previous studies demonstrated that PDAT could transfer the acyl-group from PL to synthesize TAG [19]. To confirm the acyltransferase activity of PDAT in *Aurantiochytrium* sp., it was expressed in a TAG-deficient *Saccharomyces cerevisiae* strain H1246. After galactose induction, the cells carrying PDAT could be stained with Nile red (NR), while the H1246 cells and the empty vector pYES2-carrying cells did not exhibit any staining (Fig. 1A). NR is widely used to characterize the neutral lipids [20], and thus we inferred that TAG was synthesized in cells carrying PDAT. Subsequently, thin-layer chromatography (TLC) was performed to detect the lipid profile, and as anticipated, TAG was detected in cells carrying PDAT (Fig. 1B). These results support our claim that the identified PDAT (named as *Au*PDAT) exhibits acyltransferase activity and can restore the ability of TAG biosynthesis in the TAG-deficient yeast H1246.

Endoplasmic reticulum (ER) is generally the main site of TAG assembly [19]. Once PDAT is involved in TAG biosynthesis, it may be localized in ER. However, there was no predicted signal peptide or C-terminal ER retrieval motif [21] in *Au*PDAT sequence. To confirm the localization of *Au*PDAT, it was fused with green fluorescent protein (GFP) and expressed in *Aurantiochytrium* sp. The green fluorescence was observed to co-localize with the red fluorescence from ER-tracker (Additional file 2), indicating that *Au*PDAT is located in ER. As no transmembrane domain (TMD) was identified in *Au*PDAT, we deduced *Au*PDAT resides in the ER lumen.

### Contribution of *AuPDAT* is growth stage-dependent

**Fig. 2:**
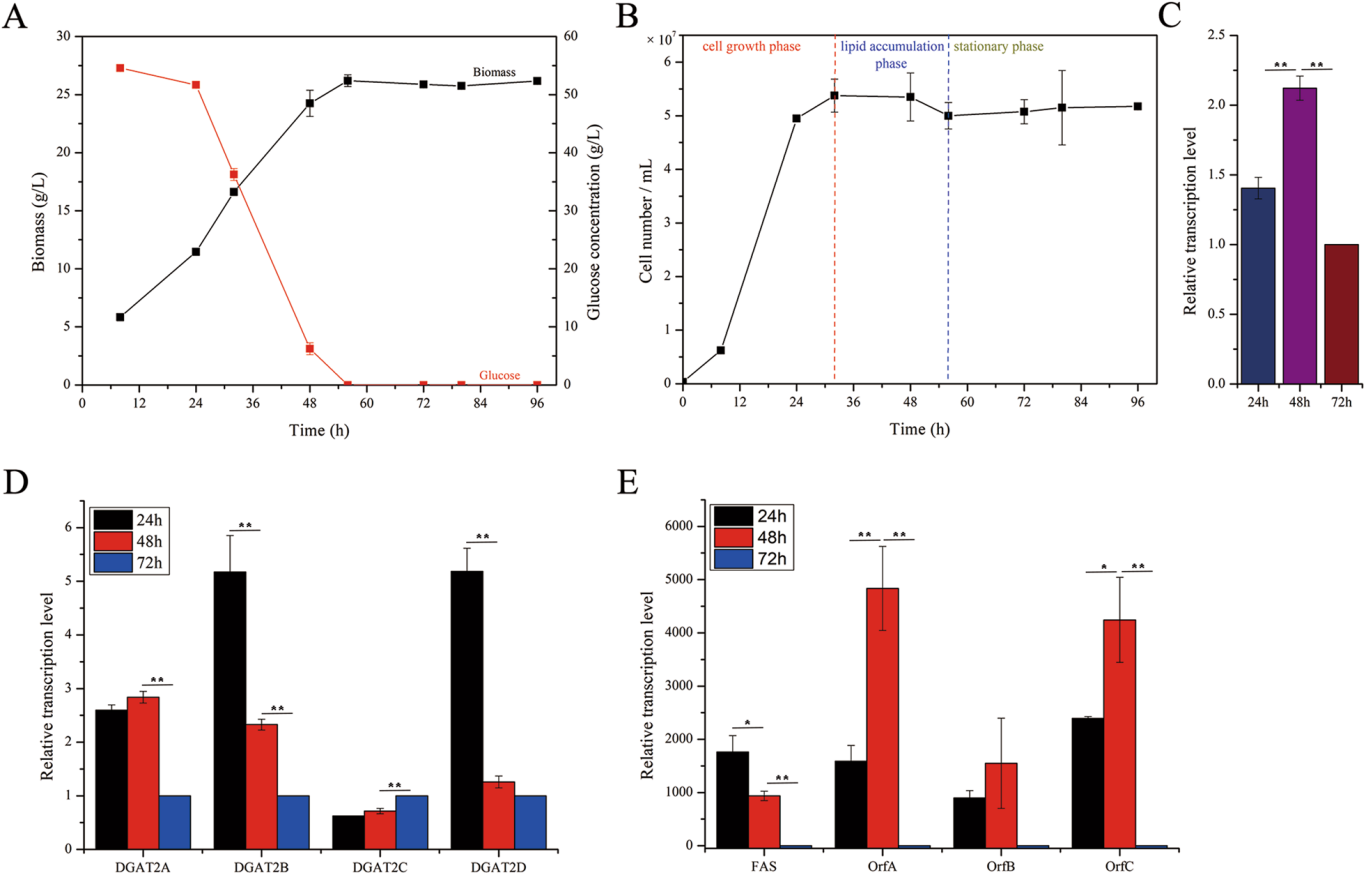
Genes involved in lipid synthesis is stage-dependent. **A** Glucose utilization and biomass accumulation at different growth-stages of *Aurantiochytrium* sp. SD116. **B** Cell number of SD116 strain at different growth-stages. The transcriptional levels of *Au*PDAT (**C**), DGAT2s (**D**), and fatty acid synthesis genes (**E**) in SD116 strain at different growth-stages

Prior to investigating the function of *Au*PDAT, growth characteristics of *Aurantiochytrium* sp. SD116 were monitored. As shown in Fig. 2A, glucose was fully utilized by SD116 after 56 h, and the maximum biomass was obtained simultaneously. However, the maximum number of cells was recorded at 32 h. Based on these results, the life cycle of *Aurantiochytrium* sp. was divided into three distinct phases: cell growth phase, lipid accumulation phase, and stationary phase (Fig. 2B). Samples obtained at 24 h, 48 h and 72 h represented each of these three phases, respectively.

Transcription analysis revealed the highest transcript abundance of *AuPDAT* was at 48 h, indicating that *Au*PDAT contributes more at the lipid accumulation phase (Fig. 2C). By contrast, DGATs displayed various transcription patterns (Fig. 2D). Four DGATs were identified in *Aurantiochytrium* sp. SD116, and our previous study showed that DGAT2C and DGAT2D account for the majority of DGAT functions, with a preference for polyunsaturated fatty acids (PUFAs) and saturated fatty acids (SFAs) as substrates, respectively [15]. Transcription analysis revealed DGAT2D contributes more at the cell growth phase while DGAT2C contributes more at the stationary phase, indicating that the process of assembling PUFAs and SFAs into TAG by Kennedy pathway was difference in *Aurantiochytrium* sp.

In *Aurantiochytrium* sp., two distinct pathways are involved in the biosynthesis of fatty acids, which includes four key genes *FAS*, *OrfA*, *OrfB*, and *OrfC*. Fatty acid synthase (FAS) is responsible for SFA synthesis and its highest transcriptional level was observed at the cell growth phase (Fig. 2E). Unlike *FAS*, the highest transcript abundance of PUFA synthase genes including *OrfA*, *OrfB*, and *OrfC* were observed at the lipid accumulation phase (Fig. 2E). Consistently, the transcription levels of all these genes decreased dramatically at the stationary phase, indicating fatty acids are mainly synthesized at cell growth phase and lipid accumulation phase. Notably, both *DGAT2D* and *FAS* showed a consistent pattern at different growth-stages, whereas DGAT2C and PUFA synthase displayed varying patterns. In addition to DGAT2C, there may be other enzymes that use PUFAs as substrates.

### AuPDAT contributes less in TAG synthesis

A linear DNA deletion cassette with *zeo*^R^ antibiotic gene was constructed to delete the *AuPDAT*. However, both the intact *AuPDAT* gene and *zeo*^R^-inserted *AuPDAT* gene were observed in the mutant cells, indicating that at least two *AuPDAT* are present in *Aurantiochytrium* sp. Fortunately, after another DNA deletion cassette with *neo*^R^ antibiotic gene was used to disrupt the other *AuPDAT*, the *AuPDAT* was not detected in the duel deletion mutant named ΔPDAT (Additional file 3), and no transcript of *AuPDAT* was detected in ΔPDAT, confirming *Au*PDAT was completely deleted (Additional file 3).

**Fig. 3:**
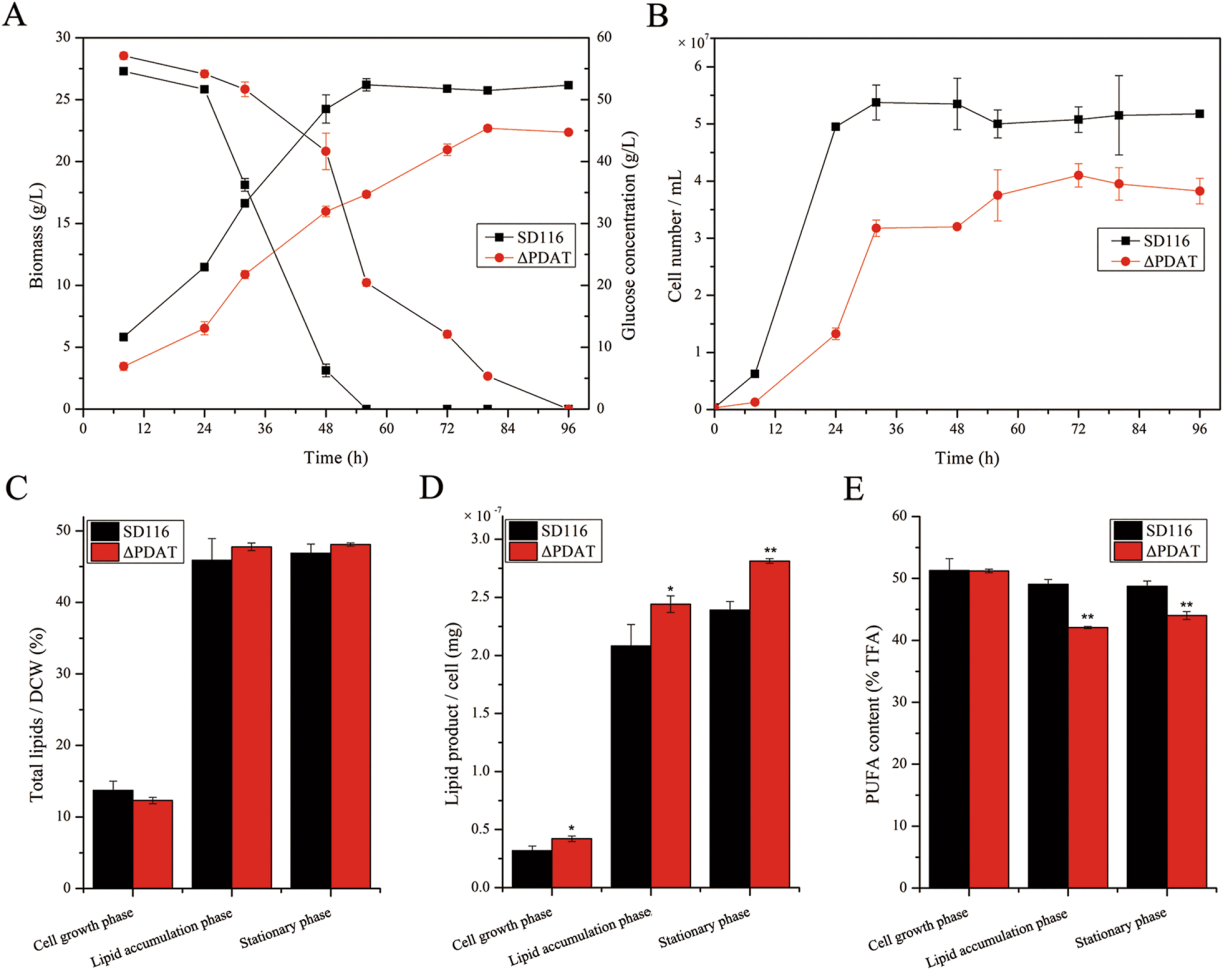
Determination of strain phenotypes of SD116 and ΔPDAT. **A** Glucose utilization and biomass accumulation at different growth-stages. **B** Cell numbers of SD116 and ΔPDAT at different growth-stages. **C** Total lipids content in DCW. **D** Lipid product in a single cell. **E** PUFA content in total fatty acids (TFA)

As shown in Fig. 3, the capacities of glucose utilization, growth rate, and final biomass of ΔPDAT were significantly lower than those of SD116. Notably, the total lipid content in ΔPDAT was comparable to that in SD116, while lipid accumulation in single cell was enhanced after disruption of *AuPDAT* (Fig. 3D). These results suggested that the primary reason for the decreased biomass of ΔPDAT was due to the lower cell number (Fig. 3B). Additionally, the PUFA content in ΔPDAT was similar to that in SD116 at the cell growth phase, but it decreased at lipid accumulation and stationary phase (Fig. 3E). Notably, the DHA content in ΔPDAT was also decreased at the cell growth phase (Additional file 4). Previous study indicated the freshly synthesized PUFAs were initially incorporated into PC and then mobilized to TAG via DAG in Thraustochytrids [16]. Disruption of *Au*PDAT may limit the process of PUFAs transfer from PC to TAG, causing a decreased PUFAs content in total fatty acids (TFA).

**Fig. 4:**
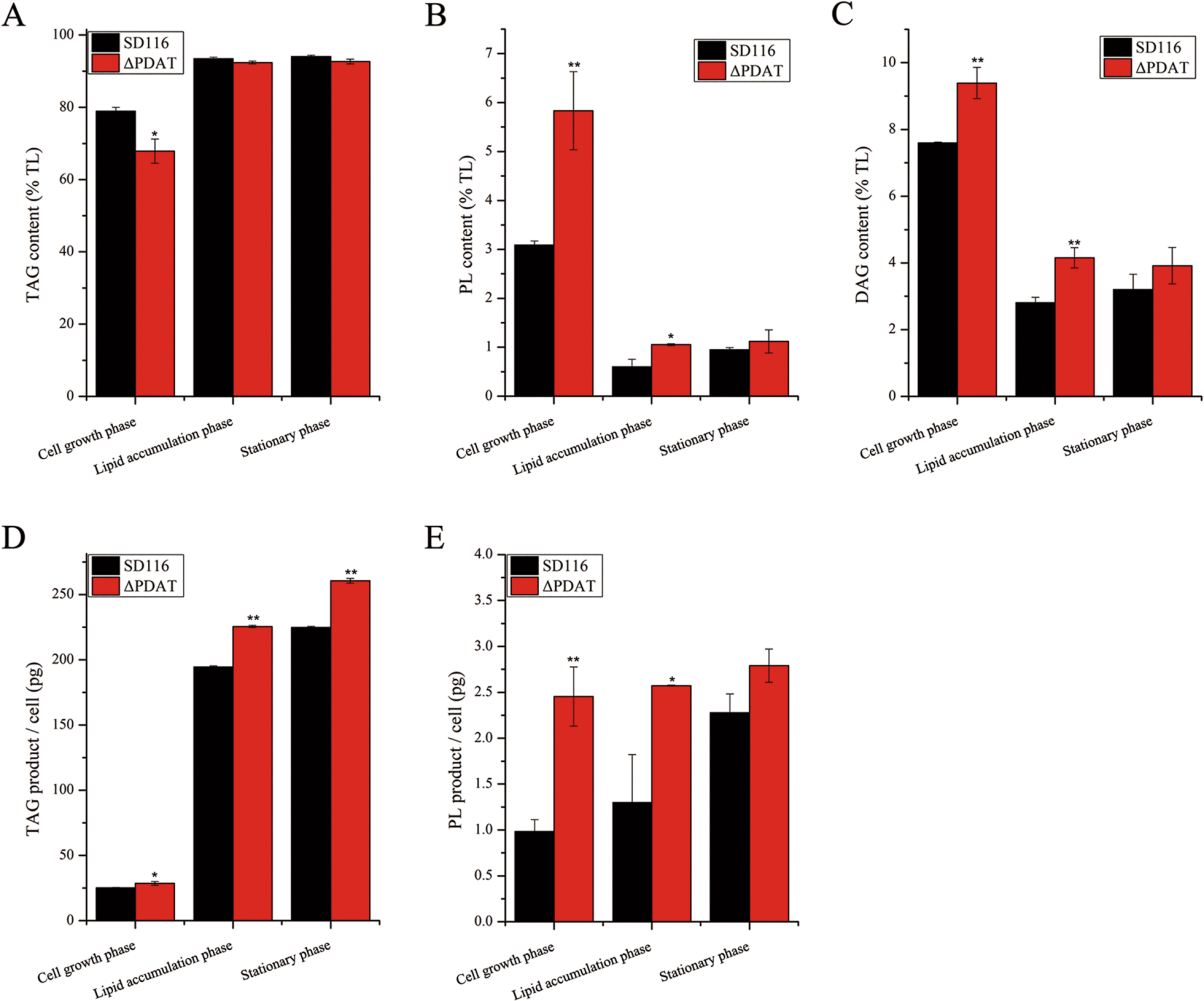
Analysis of the concentrations of TAG, PL, and DAG in SD116 and ΔPDAT. The contents of TAG (**A**), PL (**B**), and DAG (**C**) in total lipid of SD116 and ΔPDAT. The TAG product (**D**) and PL product (**E**) in a single cell of SD116 and ΔPDAT

To investigate the contribution of PDAT in TAG biosynthesis, the concentration of TAG was determined by thin-layer chromatography/flame ionization detection (TLC–FID) (Fig. 4). The TAG content in ΔPDAT was slightly lower than that in SD116 at the cell growth phase, while there was no significant difference at lipid accumulation phase and stationary phase (Fig. 4A). Specifically, the TAG product in the single cell of ΔPDAT was higher than that in SD116 (Fig. 4D). These results indicated *Au*PDAT contributes less in TAG biosynthesis. The transcripts of all DGAT2s genes, especially DGAT2A, were up-regulated in ΔPDAT compared to those in SD116 at lipid accumulation phase (Additional file 5), which may compensate for the disability of TAG biosynthesis that results from PDAT inactivation. Meantime, we found the transcript abundance of *FAS* responsible for SFA biosynthesis increased in ΔPDAT compared to that in SD116 at the lipid accumulation phase (Additional file 5), leading to a higher synthesis of SFA. The increased transcript of *FAS* may be another reason resulted in a decreased PUFAs content.

### *AuPDAT* is involved in the regulation of PL homeostasis

**Fig. 5:**
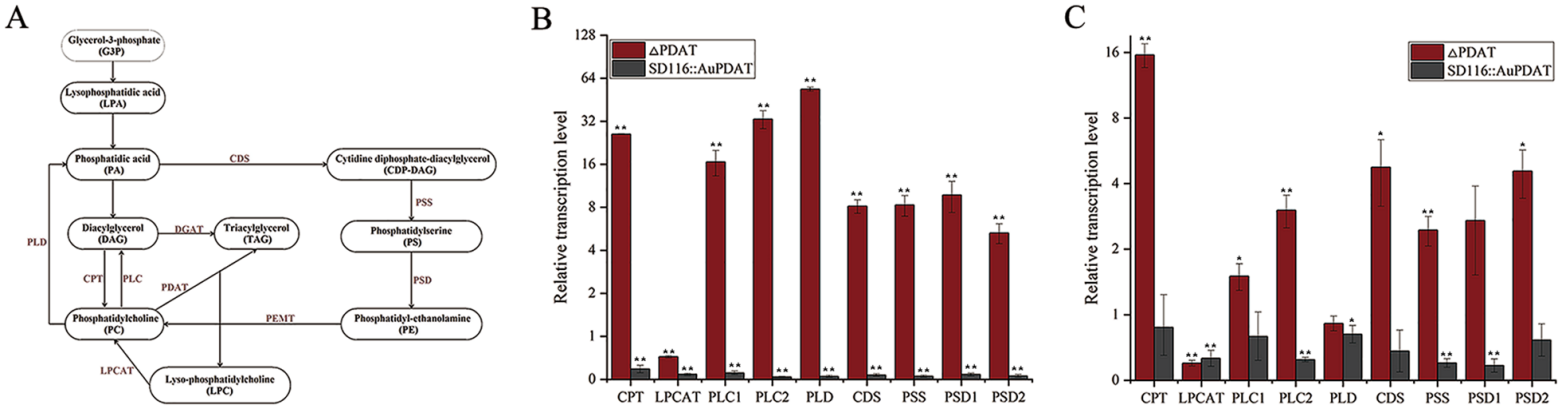
Transcriptional levels of PL synthesis-related genes. **A** Putative pathway for biosynthesis of PL in *Aurantiochytrium* sp. Transcriptional levels of PL synthesis-related genes in SD116 and ΔPDAT at the cell growth phase (**B**) and lipid accumulation phase (**C**). Note: The expression level of related genes in strain SD116 is marked as 1. *CPT* cholinephosphotransferase, *LPCAT* lyso-phosphatidylcholine acyltransferase, PEMT: phosphatidylethanolamine N-methyltransferase, *PLC* Phospholipase C, *PLD* Phospholipase D, *CDS* phosphatidate cytidylyltransferase, *PSS* phosphatidylserine synthase, *PSD* phosphatidylserine decarboxylase proenzyme

In addition to TAG, PL content in SD116 and ΔPDAT was determined. Although PL product in the single cell of ΔPDAT was accumulated at cell growth phase and lipid accumulation phase, it was comparable to that in SD116 at stationary phase (Fig. 4E). Disruption of PDAT resulted in inactivation of the pathway that transfers acyl-group from PL to synthesize TAG, which may affect the PL homeostasis. In thraustochytrids, PC is the most abundant component of PL [3]. Generally, eukaryotic PC synthesis follows the head group activation pathway, with the final and crucial step catalyzed by cholinephosphotransferase (CPT). The transcriptional level of *CPT* in ΔPDAT was found to be higher than that in SD116 (Fig. 5). CPT uses DAG and cytidine diphosphate (CDP)-choline as substrates, and the increased expression may indicate a feedback response to DAG and CDP-choline. Additionally, PC can be synthesized by phosphatidylethanolamine (PE) methylation, but a low level of PE was found in thraustochytrids. And the phosphatidylethanolamine N-methyltransferase (PEMT) encoding gene was not identified in this strain, suggesting it is not a key pathway for PC synthesis. Instead, thraustochytrids have a high level of lyso-phosphatidylcholine (LPC) [16], implying that the synthesis of PC probably follows the sequential acylation of sn-glycerol-3 phosphocholine (G3PC), with LPC acyltransferase (LPCAT) catalyzing the final and critical step. Interestingly, in contrast to CPT expression, transcriptional level of LPCAT in ΔPDAT was decreased compared with that in SD116 (Fig. 5). This may be due to feedback inhibition resulting from the accumulation of PL. Significantly, the transcripts of genes related to PC turnover (Phospholipase C (PLC1 and PLC2), Phospholipase D (PLD)) were up-regulated at the cell growth phase (Fig. 5B), which may be induced by the accumulated PC content. In addition, the expression level of PE synthesis genes including phosphatidate cytidylyltransferase (CDS), phosphatidylserine synthase (PSS), and phosphatidylserine decarboxylase proenzyme (PSD1 and PSD2) were enhanced compared with those in SD116 (Fig. 5). PDAT possesses broad substrate activities toward various PLs including PC, PE, and PS. Disruption of *Au*PDAT may disturb the PL homeostasis, further affect their metabolic pathway.

**Fig. 6:**
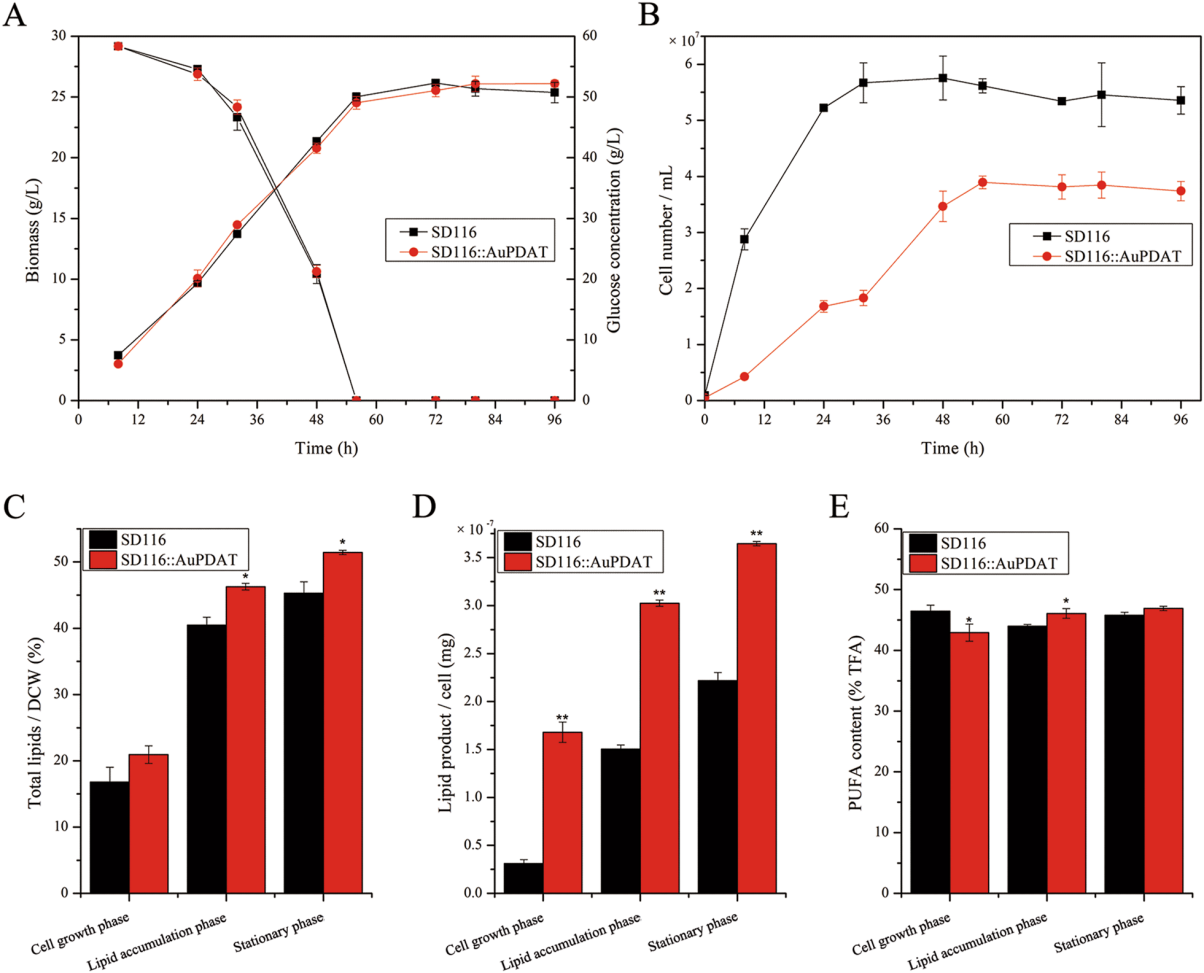
Determination of strain phenotypes of SD116 and SD116::*Au*PDAT. **A** Glucose utilization and biomass accumulation at different growth-stages. **B** Cell numbers of SD116 and SD116::*Au*PDAT at different growth-stages. **C** Total lipids content in DCW. **D** Lipid product in a single cell. **E** PUFA content in total fatty acids (TFA)

**Fig. 7:**
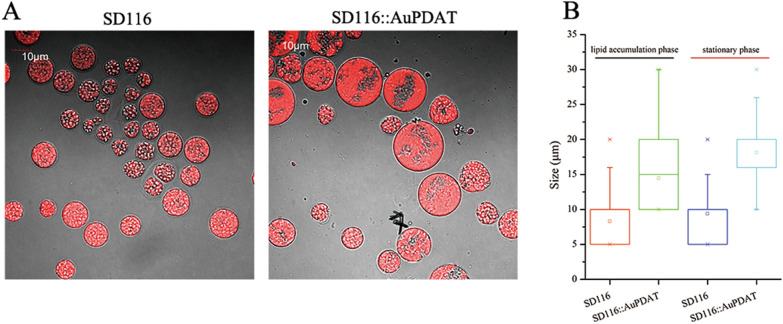
Cell diameters of SD116 and SD116::*Au*PDAT. **A** Images of SD116 and SD116::*Au*PDAT at stationary phase. **B** Cell diameter of SD116 and SD116::*Au*PDAT at lipid accumulation phase and stationary phase

To further study the function of *AuPDAT*, *AuPDAT* was overexpressed in SD116. The transcriptional levels of *AuPDAT* in *Au*PDAT overexpressed strain (SD116::*Au*PDAT) were 89-fold and 69-fold higher than those in SD116 at the cell growth phase and lipid accumulation phase, respectively (Additional file 6), indicating successful overexpression of *AuPDAT* in SD116::*Au*PDAT. Glucose utilization and cell growth were subsequently assessed, and it was observed that glucose utilization and cell biomass in SD116::*Au*PDAT were similar to those in SD116 (Fig. 6A). However, the cell growth rate and final cell number of SD116::*Au*PDAT were significantly lower than those of SD116 (Fig. 6B). More lipids were accumulated in cells of SD116::*Au*PDAT (Fig. 6D), and the cell diameter of SD116::*Au*PDAT was larger than that of SD116 (Fig. 7). These results indicated that overexpression of *AuPDAT* led to a suppressive effect on cell division. Notably, there was no significant difference in cell diameter between ΔPDAT and SD116 (data not shown), but ΔPDAT had a lower cell count than SD116, possibly due to the disruption of PL homeostasis. The transcriptional level of genes related to PL synthesis and turnover were determined in SD116::*Au*PDAT. And results showed all the transcription levels of these genes in SD116::*Au*PDAT were down-regulated (Fig. 5). Except for LPCAT and PLD, genes related to PL metabolism showed opposite expression trends between ΔPDAT and SD116::*Au*PDAT (Fig. 5), suggesting function of *Au*PDAT is involved in the regulation of PL homeostasis. In addition, we found the PUFA and DHA contents in SD116::*Au*PDAT were similar to those in SD116 at the stationary phase (Fig. 6 and Additional file 7). Based on all these results, we inferred *Au*PDAT mainly acted as a regulator in lipid metabolism.

## Discussion

There are two distinct pathways for TAG biosynthesis in nature, and almost all the genes involved in these pathways had been predicted in thraustochytrids [22, 23]. In our recent study, we found four DGATs, which are the key and rate-limiting enzymes in Kennedy pathway, in thraustochytrid *Aurantiochytrium* sp. SD116. Overexpression of these DGATs resulted in improved TAG production, indicating the effectiveness of the Kennedy pathway [15]. In this study, a candidate PDAT belonging to acyl-CoA independent TAG biosynthesis pathway was identified in the genome of *Aurantiochytrium* sp. SD116, while it is distantly related to PDATs found in plant and most fungus (Additional file 1). In order to confirm the enzymatic activity of this predicted PDAT (*Au*PDAT), it was first expressed in TAG-deficient *S. cerevisiae* strain H1246. The restored ability of TAG synthesis in H1246 implied *Au*PDAT had the acyltransferase activity. Subsequently, *Au*PDAT were completely disrupted in *Aurantiochytrium* sp., and result in an accumulated PL. These results indicated the conversion of PL to TAG was impaired due to the inactivity of *Au*PDAT. Based on the ex vivo and in vivo experiments, we confirmed the PDAT-mediated TAG synthesis was effective and intact.

Compared to the well-studied Kennedy pathway, there are only a few reports on the PDAT-mediated pathway in microorganisms [5]. What is the role of the acyl flux from PL to TAG? In this study, we found that complete disruption of *Au*PDAT led to limited rates of cell growth and glucose utilization. The final cell number and biomass were also decreased in ΔPDAT compared to SD116. However, the total lipid content did not differ significantly between the two strains, suggesting that the PDAT-mediated pathway may play a minor role in TAG biosynthesis. Interestingly, the total lipid and TAG contents in cells of ΔPDAT were even higher than those of SD116. Additionally, lipid profile revealed both of PL and TAG in cell of ΔPDAT were higher compared to SD116. Besides forming the cellular membranes, PLs are essential for vital cellular processes, such as serving as a reservoir of second messengers, providing precursors for the synthesis of macromolecules, and function as molecular chaperones [24]. The changed PLs concentration may disturb the cellular membranes structure and vital cellular processes, which could explain the limited cell growth observed in ΔPDAT and SD116:: *Au*PDAT. PL dynamics was observed in both ΔPDAT and SD116:: *Au*PDAT, and the transcriptional levels of most genes related to PL synthesis and turnover were changed. Because the PL metabolism pathway was complex, it is difficult to confirm the process of cellular PL homeostasis. Here, we found that the expressional trends of genes related to PL metabolism were opposite in both mutant strains, suggesting that the role of *Au*PDAT may be to regulate PL homeostasis in *Aurantiochytrium* sp. to maintain the cell physiological processes.

The migration of fatty acids from PL to TAG during the fermentation cycle was observed in previous reports [3, 16]. Whether *Au*PDAT identified in this study catalyzes this process? Previous study reported PC-20:5/22:6 and PC-22:6/22:6 contributed to half of the amount of total PC at the cell growth phase, while the PUFAs in PC decrease obviously in the lipid accumulation phase [3]. In this study, we found that the PUFA content in total fatty acids was slightly reduced in ΔPDAT compared to SD116, and *Au*PDAT contributes more at the lipid accumulation phase. Thus, we deduced *Au*PDAT could use PUFA as the substrate to incorporate into TAG. At the cell growth phase, most of the synthesized PUFAs preferentially incorporated into PL [3, 16], and the excess PLs may migrate into TAG catalyzed by *Au*PDAT. In thraustochytrids, low temperature enhanced the DHA accumulation [23, 25], which could remodel membranes to response to cold stress by improving the fluidity and integrity of the membrane. Our previous study found the expression of *Au*PDAT was depressed at this condition [26], which may result in the PL accumulation. This finding was consistent with the changes observed in the PL components at low temperatures. Here, we found PL homeostasis was related to cell growth rate and single-cell lipid production. Therefore, regulation of *Au*PDAT expression at different stages was able to tailor engineering strains with various growth rates and lipid production.

In addition to PL, TAG was synthesized along with the cells grew, especially, at the stage of lipid accumulation. Based on our results, the Kennedy pathway has been considered as the major pathway of TAG biosynthesis. According to the transcriptional profiles of DGAT2s, the mechanism of TAG biosynthesis was deduced. DGAT2C and DGAT2D have been proved to be the major enzymes for PUFAs and SFAs accumulation in the sn-3 position of TAG molecules, respectively. The expression level of DGAT2C was higher at lipid accumulation and stationary phase compared to the cell growth phase. Hence, we can deduce that PUFAs were mainly incorporated into PC at the cell growth phase, while more PUFAs were incorporated into TAG by the Kennedy pathway or transformation of PC-PUFAs to synthesize TAG catalyzed by *Au*PDAT at the lipid accumulation phase. Furthermore, DGAT2D exhibited the highest level of expression at cell growth phase, suggesting SFAs was primarily added into TAG through the Kennedy pathway at cell growth phase.

## Conclusions

An acyl-CoA independent TAG biosynthesis pathway mediated by *Au*PDAT has been confirmed to be complete and effective in thraustochytrid *Aurantiochytrium* sp. Although *Au*PDAT is capable of transferring acyl-groups from PL to DAG to synthesize TAG, it is not its primary function. Instead, *Au*PDAT appears to primarily regulate PL homeostasis, which in turn affects cell structure and activity. In addition, assembly of PUFA into TAG occurs in two stages: first, during the cell growth phase, PUFA is incorporated into PL, and then in the lipid accumulation phase, any excess of PL was transferred into TAG by *Au*PDAT. The investigation of *Au*PDAT in *Aurantiochytrium* sp. provides further insights into lipid biosynthesis and offers the possibility of creating a “customized cell factory” for lipid production.

## Methods

### Strains and culture conditions

Strains used in this study are shown in Table 1. *Aurantiochytrium* sp. SD116 was isolated in the previous study [27], and grown on the GYS medium (60 g/L glucose, 20 g/L yeast extract, 15 g/L sea salt) at 25 °C with shaking at 200 rpm. *Escherichia coli* DH5α was grown in Luria–Bertani (LB) medium at 37 °C with shaking at 160 rpm. *S. cerevisiae* strain was grown on YPD medium at 30 °C with shaking at 200 rpm. Antibiotics were used at the following concentrations: ampicillin, 100 μg/mL; zeocin, 100 μg/mL.

**Table 1:** Strains and plasmids used in this study

Strain or plasmid	Description	References or source
Strains		
*Escherichia coli* DH5α	Strain used for plasmid construction	Invitrogen
*Aurantiochytrium* sp. SD116	Wild type	[27]
ΔPDAT	Disruption of two *Au*PDAT in SD116	This study
ΔPDAT1	Disruption of one *Au*PDAT in SD116	This study
SD116::*Au*PDAT	Overexpression of *Au*PDAT gene in SD116	This study
*Saccharomyces cerevisiae*		
H1246	The TAG-deficient quadruple of *S. cerevisiae*	[29]
H1246-pYES2	H1246 harboring the empty plasmid pYES2	[29]
H1246-pDGA1	H1246 expressing yeast DGA1 gene	[29]
H1246-pPDAT	H1246 expressing *Au*PDAT gene	This study
Plasmids		
pWKZ	Plasmid carrying the *zeo*^R^ gene expression cassette	This study
pMDZ-GFP	Plasmid carrying the *GFP* and *zeo*^R^ gene co-expression cassette and 18 s homologous arm H1 and H2	[30]
pMDZ-GFP-PDAT	Plasmid carrying the *GFP* and *AuPDAT* gene co-expression cassette	This study
pWKZ-△PDAT	Plasmid carrying the *zeo*^*R*^ gene expression cassette and *AuPDAT* homologous arm H1 and H2	This study
pWKN-△PDAT	Plasmid carrying the *neo*^*R*^ gene expression cassette and *AuPDAT* homologous arm H1 and H2	This study
pYES2-PDAT	Plasmid carrying the *URA3* gene expression cassette and the *AuPDAT* gene expression cassette	This study

### Bioinformatic analysis

Conserved domains were performed by SMART program (http://smart.embl-heidelberg.de/). Predication of protein transmembrane regions was checked by the TMHMM program (http://www.cbs.dtu.dk/services/TMHMM). Phylogenetic analyses were carried out using Mega 5.0 and trees were constructed by using neighbor-joining method.

### Plasmid construction

All primers used are listed in Additional file 8. The plasmid pMDZ-GFP-PDAT was constructed based on previously constructed plasmid pMDZ-GFP [30]. pMDZ-GFP includes 18 s rDNA homologous fragment, *gfp* and zeocin with 2A-linked co-expression cassette. To construct the pMDZ-GFP-PDAT plasmid, *AuPDAT* gene was amplified from the genome of *Aurantiochytrium* sp. SD116 using the primer pair PDAT-F/PDAT-R and then seamlessly assembled with the line vector, which was amplified from pMDZ-GFP using the primer pair GFP-F/PEF1-R.

To construct the pYES2-PDAT plasmid, *AuPDAT* gene was amplified from the genome of *Aurantiochytrium sp.* SD116 using the primer pair YPDAT-F/YPDAT-R and then seamlessly assembled with the line vector, which was amplified from pYES2-DGAT2C [15] using the primer pair CYS-F/GAL-R. *Au*PDAT expression cassette contained the GAL1 promoter, the *AuPDAT* gene, and the cytochrome C terminator.

Zeocin resistance gene (*zeo*^R^) expression cassette containing the elongation factor EF1 α promoter (PEF1α), *zeo*^R^, and the cytochrome C terminator (cycTT) was assembled by overlap PCR and inserted into plasmid pMD19-T (Takara, Dalian, China), forming the pWKZ plasmid. To construct the pWKZ-△PDAT knockout plasmid, the upstream and downstream homologous recombinant fragments (H1 and H2) of *AuPDAT* gene are amplified from the genome SD116 and inserted into the upstream and downstream of the zeocin expression cassette from pWKZ plasmid, respectively.

Neomycin resistance gene (*neo*^R^) expression cassette containing the actin promoter (Patin), *neo*^R^, and the actin terminator (Tatin) was assembled by overlap PCR and inserted into plasmid pMD19-T (Takara, Dalian, China), forming the pWKN plasmid. Two homologous fragments of *AuPDAT* gene were amplified using the primer pair PDAT-H1-2F/PDAT-H1-2R and PDAT-H2-2F/PDAT-H2-2F and then was inserted into pWKN plasmid, resulting in the pWKN-△PDAT knockout plasmid.

### Transformation of yeasts and *Aurantiochytrium*

The expression vectors were transformed into *S. cerevisiae* strain H1246 using the LiAc/SS carrier DNA/PEG method [15]. Transformants of *S. cerevisiae* were selected on SC-URA medium without urea. Electroporation of *Aurantiochytrium sp*. SD116 was carried out according to the previous description [28]. After electroporation, cells were cultured on solid selective medium containing 10 g/L glucose, 20 g/L yeast extract, 10 g/L sea salt, and 15 g/L agar and 50 μg/mL of zeocin. The culture plates were incubated in the dark at 25 °C for 3 days, and the correct transformants were confirmed by PCR analysis.

### Biomass, glucose concentration, cell number and cell observation of *Aurantiochytrium* sp.

At different growth-stages, a 2 mL sample of cell culture was taken and cell counting was performed using a hemocytometer with a size of 16 × 25 squares. To determine the dry weight of cells (DCW), the cells was centrifuged at 3500 *g* for 3 min, and the resulting cells were freeze dried. The glucose content of the supernatant was measured using a SBA-40D biosensor.

The endoplasmic reticulum (ER) and nuclear staining were carried out using the ER staining kit (bestbio BB-441164) and DAPI staining kit (beyotime C1006), respectively. Briefly, after culturing the cells at 25 °C for 16 h, 1 ml cells was harvested and centrifuged to remove the medium. The cells were then washed once with PBS and resuspended in 100 μL of PBS, followed by the addition of 10 μL DAPI staining solution and ER-Tracker Red staining working solution. The cells were incubated at 37 °C for 15–30 min. After centrifugation to remove the supernatant, the cells were washed three times with PBS and observed using a laser scanning confocal microscope (Fluo View™ FV1000, Olympus). For confocal imaging, the fluorescence of ER, DAPI, and GFP was excited at 340, 586, and 488 nm, respectively, and emitted at 488, 616, and 507 nm.

### Quantitative real-time PCR (qRT-PCR) analysis

The extraction of total RNA was performed using the Trizol reagent (Thermo Scientific) and RNeasy Mini Kit (QIAGEN, Germany). Subsequently, cDNA was synthesized by using the Revert Aid First Strand cDNA Synthesis Kit (Thermo Scientific). The FastStart Universal SYBR Green Master (ROX) was used for PCR amplification, and the corresponding primers are identified in Additional file 8. The internal control, Actin, was used to normalize the expression levels, and for the calculation of relative abundances of various mRNA molecules, the 2-ΔΔCt method was applied.

### Lipid isolation and fatty acid composition analysis

Total lipid extraction and fatty acid composition analysis were performed as previously described. To obtain fatty acid methyl esters (FAMEs), lipids were reacted with 2% sulfuric acid/methanol (v/v) at 85 °C for 2.5 h. The resulting FAMEs were extracted in hexane and analyzed by gas chromatography (Agilent Technologies, 7890B) with a HP-INNOWAX capillary column (30 m × 0.25 mm, 0.25 μm film thickness). The oven temperature was programmed to increase from 100 °C to 250 °C at a rate of 15 °C per minute and then held at 250 °C for 5 min. The split ratio was 19:1 and nitrogen was used as the carrier gas. Peaks were detected using a flame ionization detector and the authentic FAME standards were used for identification.

### Analysis of lipid composition by thin-layer chromatography/flame ionization detection

The total lipids extracted as described above were diluted with chloroform to prepare a 20 mg/mL solution. The prepared solution was filtered through an organic phase filter with a pore size of 0.22 μm, and 5 μL of the sample was dotted at the origin of a chromatography rods pre-activated and equilibrated with a humidified solvent. After spotting, the chromatography strip was dried in a drying oven. The dried rods was then put into the first spreading solvent—benzene:chloroform:acetic acid (150:60:2) until the spreading front extended to a distance of 11 cm from the origin, and then taken out and dried in a drying oven with a temperature of 60 °C for 2 min. Next, the rods was transferred to the second spreading solvent (benzene:n-hexane = 1:1) until the developing front extended to a distance of 6.5 cm from the origin. The rods were then taken out and dried for another 2 min in a drying oven. After drying, the chromatography rods was put into a rod thin-layer chromatograph (IATROSCAN MK-6S), operated with an air flow rate of 2 L/min and a hydrogen flow rate of 160 mL/min. The chromatograms were integrated and the results were calculated.

### Supplementary Information

**Additional file 1: Fig.S1.** Phylogenetic analysis of PDAT in *Aurantiochytrium* sp. SD116 by the Neighbor-Joining (NJ) method.

**Additional file 2: Fig.S2.** Subcellular localization of *Au*PDAT in *Aurantiochytrium* sp. SD116.

**Additional file 3: Fig.S3.** Disruption of *Au*PDAT in *Aurantiochytrium* sp. SD116. (A) Genomic PCR detection of *Au*PDAT in SD116, ΔPDAT1, and ΔPDAT. (B) Relative transcription level of the *Au*PDAT in SD116, ΔPDAT1, and ΔPDAT. SD116: parent strain; ΔPDAT1: deletion of one *Au*PDAT, ΔPDAT: deletion of two *Au*PDAT.

**Additional file 4: Fig.S4.** DHA content in SD116 and ΔPDAT at different stages.

**Additional file 5: Fig.S5.** Transcriptional levels of DGAT2s (A) and fatty acid synthesis genes (B) in SD116 and ΔPDAT. Note: The expression level of related genes in strain SD116 is marked as 1.

**Additional file 6: Fig.S6.** Relative transcription level of *Au*PDAT in SD116::*Au*PDAT compared to that in SD116.

**Additional file 7: Fig.S7.** DHA content in SD116 and SD116::* Au*PDAT at different stages.

**Additional file 8: Table S1.** Primers used in this study.

## Data Availability

All data generated or analyzed during this study are included in this published article and its Additional files 1, 2, 3, 4 and 5.

## Abbreviations

PDAT: Phospholipid:diacylglycerol acyltransferase
TAG: Triacylglycerol
SFA: Saturated fatty acid
PUFA: Polyunsaturated fatty acid
DHA: Docosahexaenoic acid
GPAT: Acyl-CoA:glycerol-sn-3-phosphate acyl-transferase
LPAT: Lysophosphatidate acyl-transferase
PAP: Phosphatidic acid phosphatase
DGAT: Diacylglycerol acyltransferase
DAG: Diacylglycerol
LB: Luria–Bertani
TLC: Thin-layer chromatography
PL: Phospholipids
PC: Phosphatidylcholine
PE: Phosphatidylethanolamine
LCAT: Lecithin:cholesterol acyltransferase
NR: Nile Red
ER: Endoplasmic reticulum
GFP: Green fluorescent protein
TMD: Transmembrane domain
CPT: Cholinephosphotransferase
CDP: Cytidine diphosphate
LPC: Lyso-phosphatidylcholine
PEMT: Phosphatidylethanolamine n-methyltransferase
PLC: Phospholipase C
PLD: Phospholipase D
CDS: Phosphatidate cytidylyltransferase
PSS: Phosphatidylserine synthase
PSD: Phosphatidylserine decarboxylase proenzyme
G3PC: Sn-glycerol-3 phosphocholine
LPCAT: LPC acyltransferase
DCW: Dry cell weight
