# Supplementary figures and images for: A phospholipid:diacylglycerol acyltransferase is involved in the regulation of phospholipids homeostasis in oleaginous Aurantiochytrium sp
Source: Biotechnol Biofuels Bioprod. 2023 Sep 27;16:142. doi: 10.1186/s13068-023-02396-y (PMC10523756; doi:10.1186/s13068-023-02396-y)

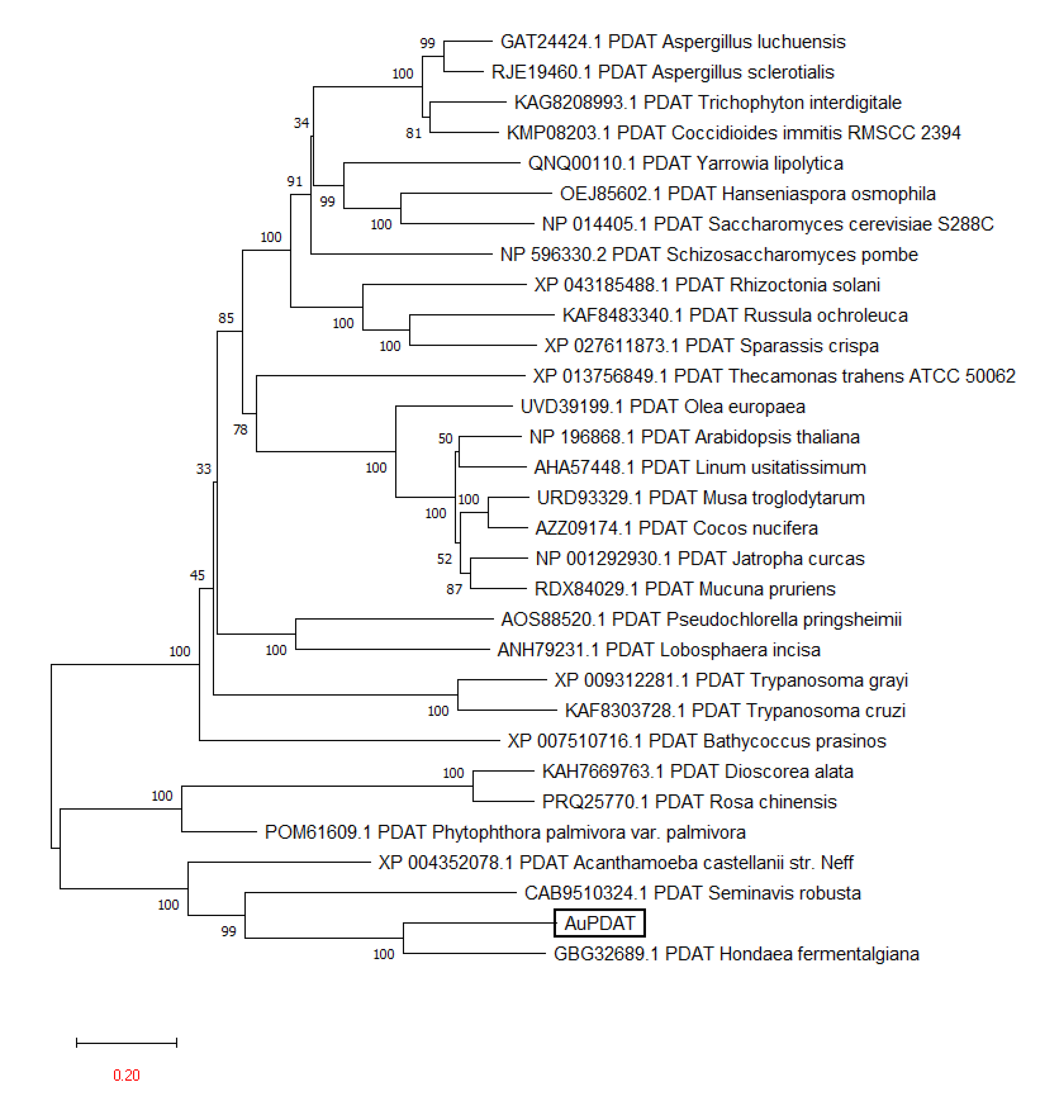

Supplement: Supplementary file 1 — Additional file 1: Fig.S1. Phylogenetic analysis of PDAT in Aurantiochytrium sp. SD116 by the Neighbor-Joining (NJ) method. [file 13068_2023_2396_MOESM1_ESM.tif]

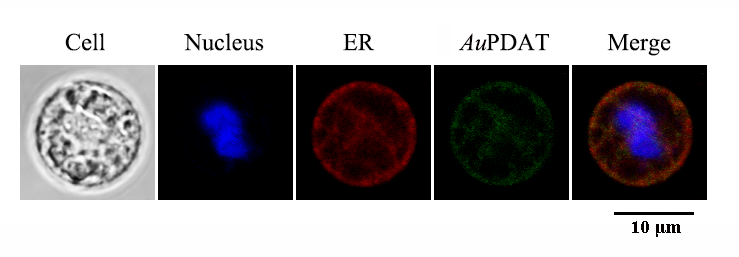

Supplement: Supplementary file 2 — Additional file 2: Fig.S2. Subcellular localization of AuPDAT in Aurantiochytrium sp. SD116. [file 13068_2023_2396_MOESM2_ESM.tif]

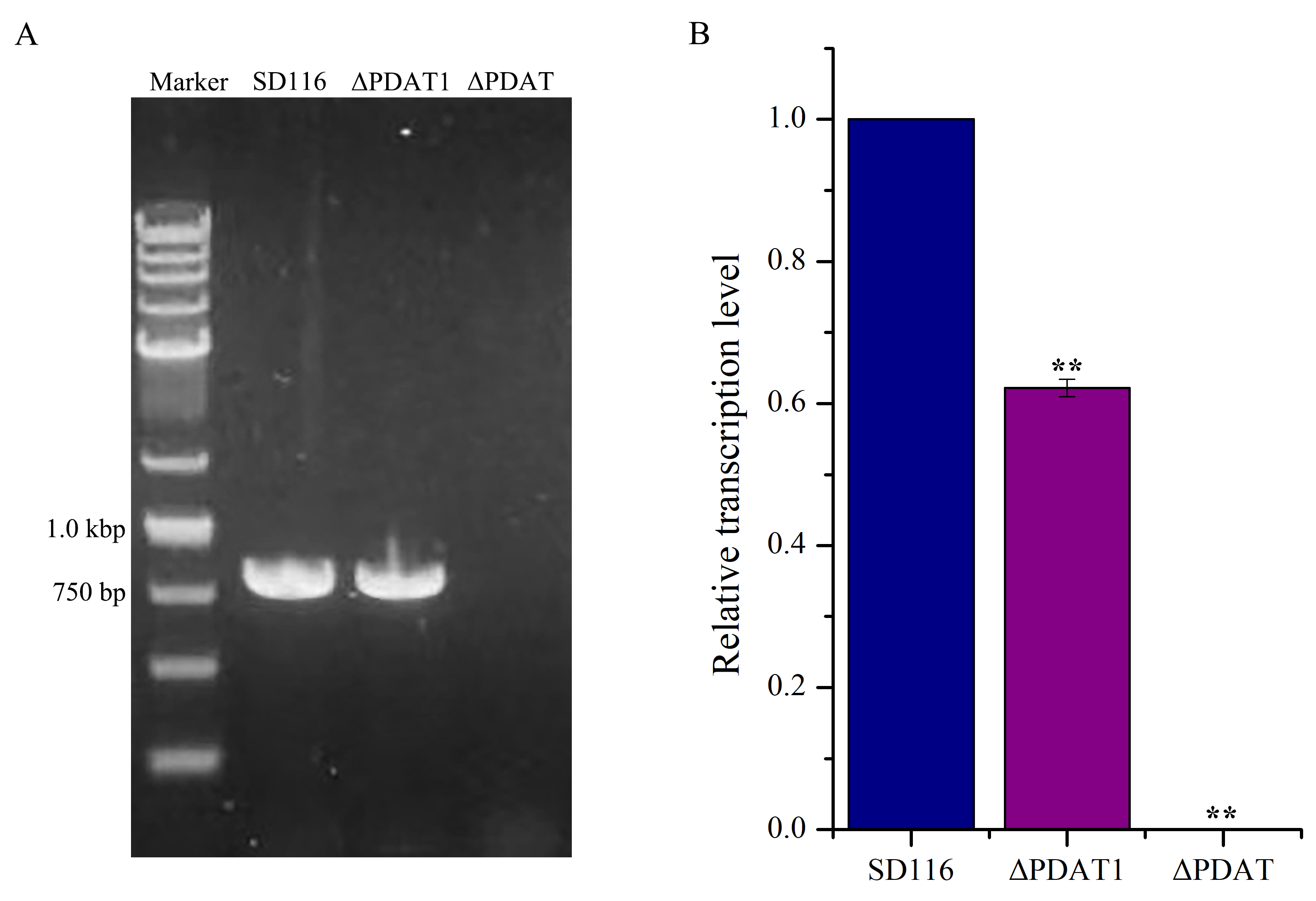

Supplement: Supplementary file 3 — Additional file 3: Fig.S3. Disruption of AuPDAT in Aurantiochytrium sp. SD116. (A) Genomic PCR detection of AuPDAT in SD116, ΔPDAT1, and ΔPDAT. (B) Relative transcription level of the AuPDAT in SD116, ΔPDAT1, and ΔPDAT. SD116: parent strain; ΔPDAT1: deletion of one AuPDAT, ΔPDAT: deletion of two AuPDAT. [file 13068_2023_2396_MOESM3_ESM.tif]

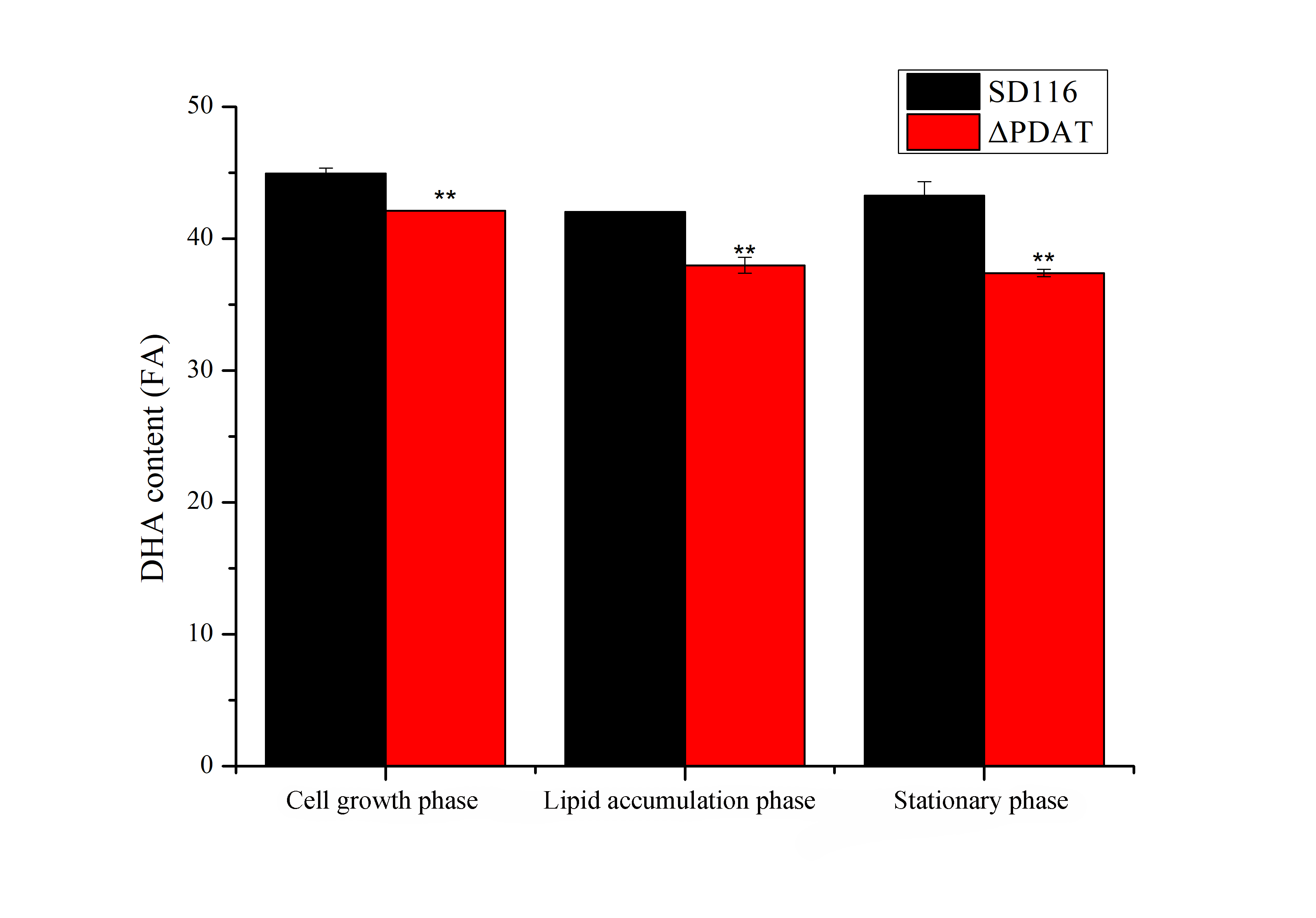

Supplement: Supplementary file 4 — Additional file 4: Fig.S4. DHA content in SD116 and ΔPDAT at different stages. [file 13068_2023_2396_MOESM4_ESM.tif]

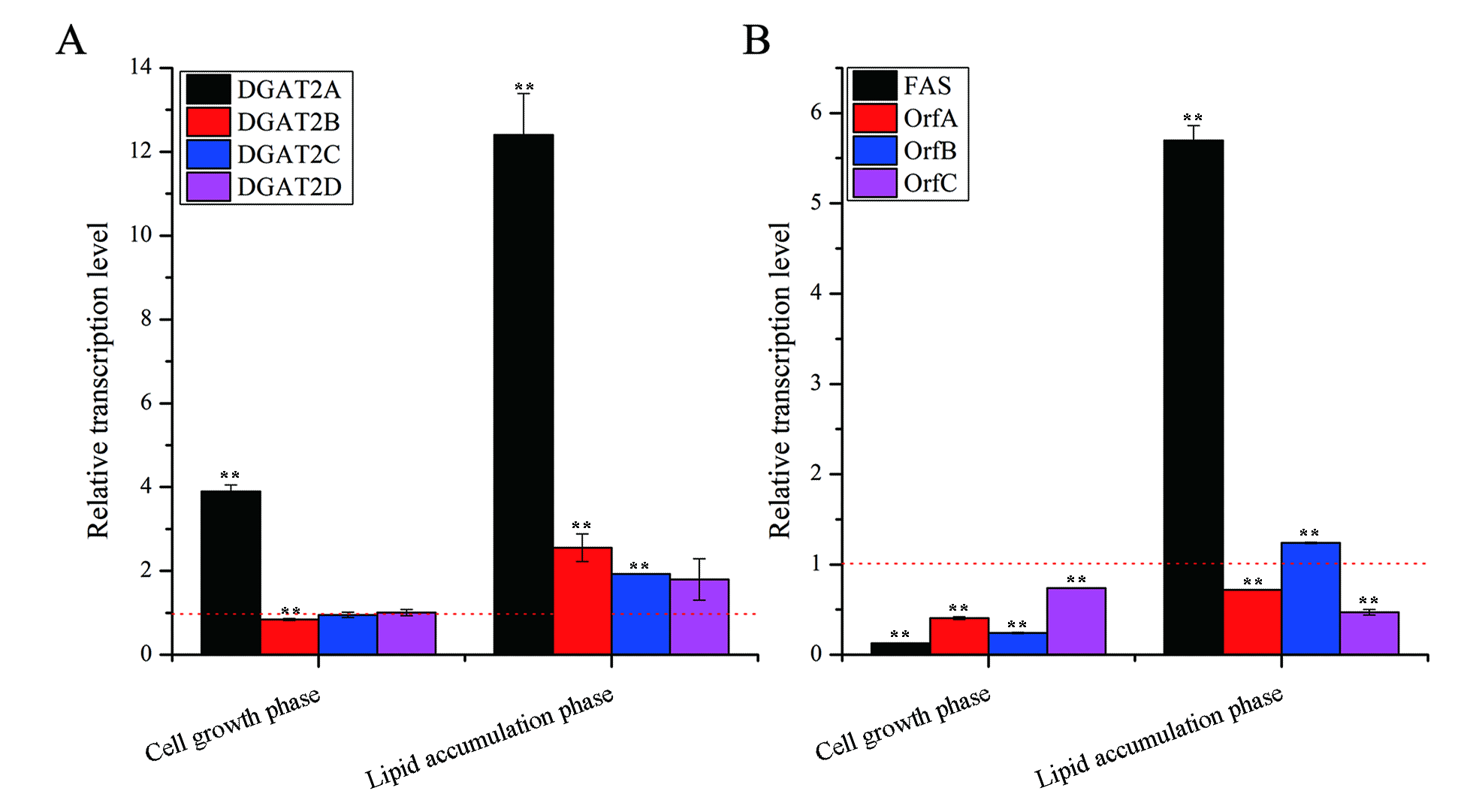

Supplement: Supplementary file 5 — Additional file 5: Fig.S5. Transcriptional levels of DGAT2s (A) and fatty acid synthesis genes (B) in SD116 and ΔPDAT. Note: The expression level of related genes in strain SD116 is marked as 1. [file 13068_2023_2396_MOESM5_ESM.tif]

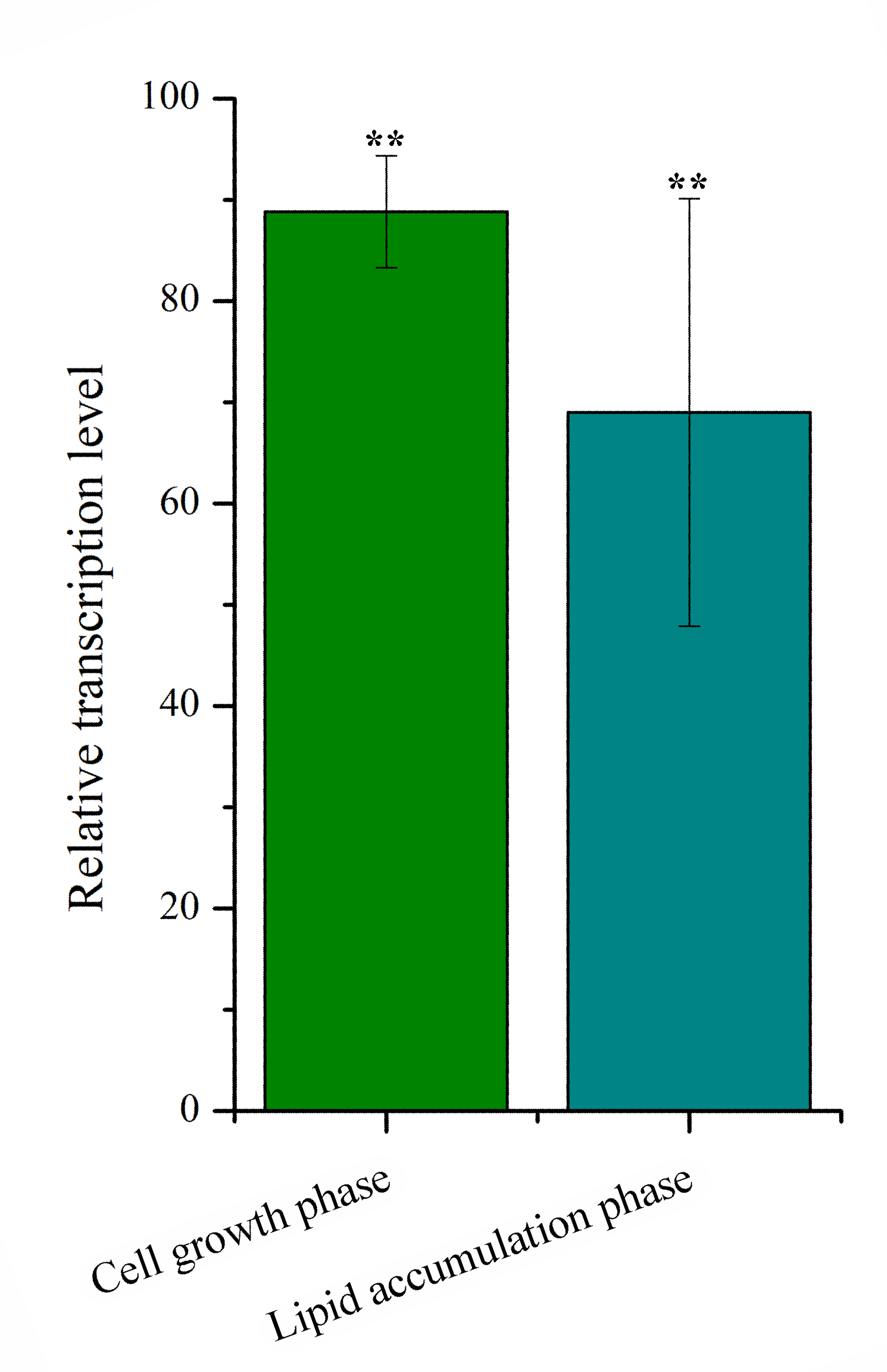

Supplement: Supplementary file 6 — Additional file 6: Fig.S6. Relative transcription level of AuPDAT in SD116::AuPDAT compared to that in SD116. [file 13068_2023_2396_MOESM6_ESM.tif]

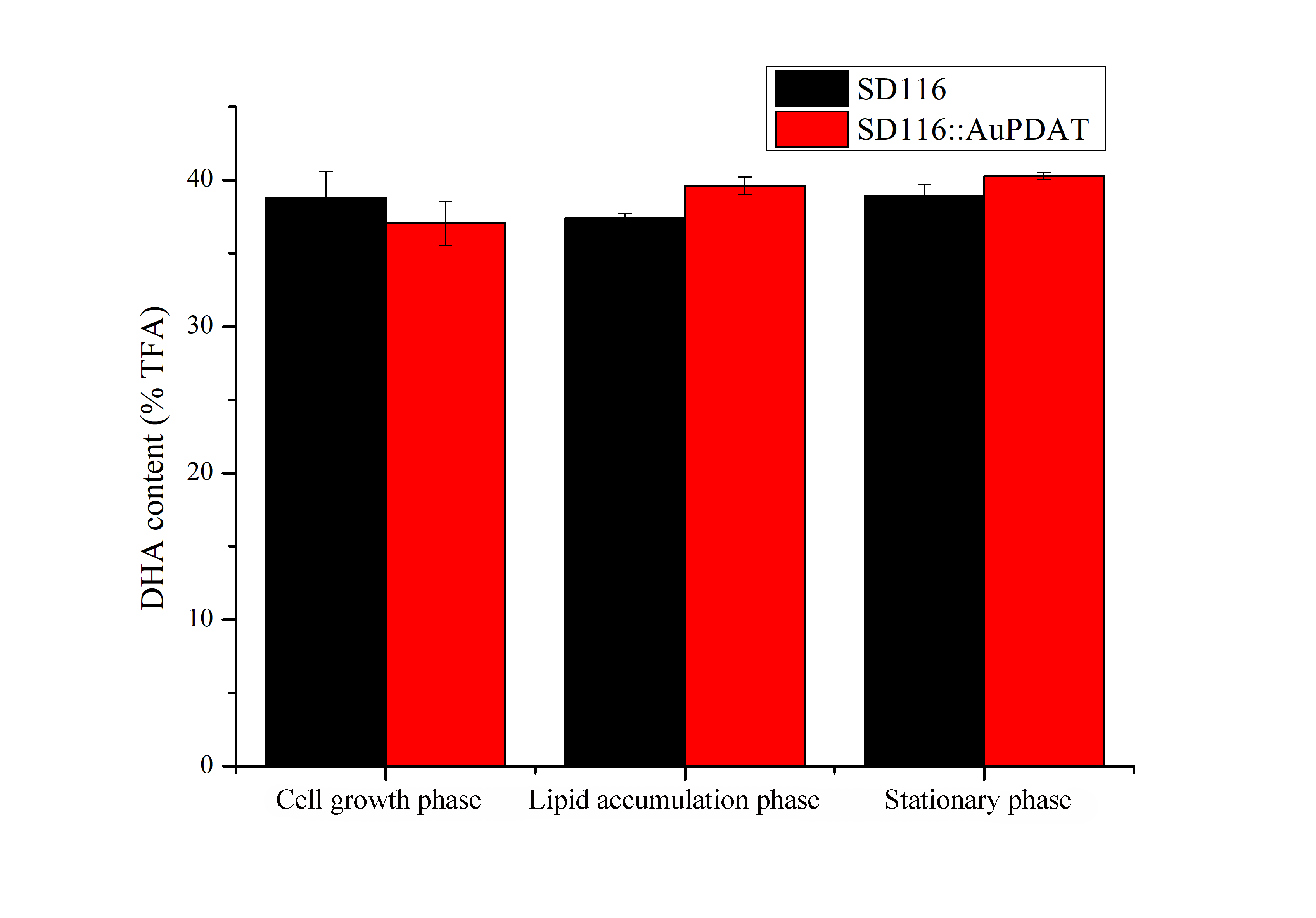

Supplement: Supplementary file 7 — Additional file 7: Fig.S7. DHA content in SD116 and SD116:: AuPDAT at different stages. [file 13068_2023_2396_MOESM7_ESM.tif]
