# Supplementary material for: A phospholipid:diacylglycerol acyltransferase is involved in the regulation of phospholipids homeostasis in oleaginous Aurantiochytrium sp
Source: Biotechnol Biofuels Bioprod. 2023 Sep 27;16:142. doi: 10.1186/s13068-023-02396-y (PMC10523756; doi:10.1186/s13068-023-02396-y)
Supplement: Supplementary file 8 — Additional file 8: Table S1. Primers used in this study. [file 13068_2023_2396_MOESM8_ESM.docx]

**Table S1.** Primers used in this study.

| Primer | Sequence |
| --- | --- |
| PDAT-F | GAAGCTACTAGCACCAAACAAAATGGAGAGGAATGCTGGCTCAGT |
| PDAT-R | CGACATTCCTCCTCCTCCTCCTCCTTCGGCTAGAGGGGCACTATAGC |
| GFP-F/ | GGAGGAGGAGGAGGAGGAATGTCGAAG |
| PEF1-R | TTTGTTTGGTGCTAGTAGCTTCGAAC |
| YPDAT-F | AGGGAATATTAAGCTTATGGAGAGGAATGCTGGCTC |
| YPDAT-R | GCCCTCTAGATGCATGTTCGGCTAGAGGGGCACTAT |
| CYS-F | CATGCATCTAGAGGGCCGCATCATG |
| GAL-R | AAGCTTAATATTCCCTATAGTGAGT |
| PDAT-H1-1F | ATCGCTATAATGACCCCGAAGCAAAGACGTTGATGATGATGTC |
| PDAT-H1-1R | GTTGTTGGACGGTGGATTGCATGTTTGAACCTCCAATCAAGTTTCTG |
| PDAT-H2-1F | CTCGAAGGCTTTAATTTGCAAAGTAAAGTGATGTATATTGC |
| PDAT-H2-1R | GTCGACGAATTAGCCATGGTCTTGCTGTTCGTGTTCGTCAGGGC |
| PDAT-H1-2F | AATTCGGGGCGGCCGCGTACCGTACTCAGCGTTGAT |
| PDAT-H1-2R | CAGCCTACACGGTACCTTCTTCATCTTGATTTGCTC |
| PDAT-H2-2F | GGAGGATATTCATATGAACCCTGAGAGCTCGCAATAT |
| PDAT-H2-2F | GAGCTCTACGTGTCGACCAAGAGGAAATTGAGAATAT |
| Actin-F | TGGACCAGAAGGACGCCTAT |
| Actin-R | TGACACCATCACCAGAGTCG |
| DGAT2A-F | AGGAGACTCGCACAAGGTT |
| DGAT2A-R | TAGCAAACGGCATCACAC |
| DGAT2B-F | TGAGACGAGGTTGGATGTTC |
| DGAT2B-R | CAATGAATGGGATGGTTGAC |
| DGAT2C-F | TCGTGGTCATTGGCATAG |
| DGAT2C-R | GTCTGTGGCTTTGTTATCCTC |
| DGAT2D-F | TGCCGATGATTATGGTAGTC |
| DGAT2D-R | CAATGCCTGGAAACTTGC |
| PDAT-F | CGTGTTCGGACCTCTTATCAA |
| PDAT-R | GCGGACCACCAATAGGAAT |
| OrfA-F | AGTCCAGCCACGAGTTCTAC |
| OrfA-R | AACCTTGACGGCGATGAG |
| OrfB-F | CCTCGGTCCTCTTCACTACTC |
| OrfB-R | AATGGCACCACCTTCACC |
| OrfC-F | CAAGGAGATGGGCTACGAT |
| OrfC-R | GGATGTGCTCAGGGATGG |
| FAS-F | GCATCTACCACCGTCTTGTTG |
| FAS-R | GGAGCAGAACCAGTCACCTT |
| CPT-F | GCTGCTGCTGAAATGTTG |
| CPT-R | GCAAAGTCCAAGACCAATG |
| LPCAT-F | TTGTTGTAGCACCGCACTC |
| LPCAT-R | ATGAGAAGTTGGCGATGC |
| PLC1-F | GCACAGGAAAGGTGGAATG |
| PLC1-R | TGGGTTCTTCAGAGATGCC |
| PLC2-F | TGATGGAGGAGAAAGAGGC |
| PLC2-R | TCGCATAGTGCTTGAAGTG |
| PLD-F | CGAACGATGATGCCAAGAG |
| PLD-R | ACGAGATGTAACCAGGTCCG |
| CDS-F | AACCAATCTGCGGACTCTG |
| CDS-R | CCCATCTGTTGAAGGTAAACC |
| PSS-F | TAACTGGACTGGTCGTCGTG |
| PSS-R | TGGCTCTTGATGGGAATG |
| PSD1-F | AATCAAATCCAGGAGCACC |
| PSD1-R | AGGCAAGGACTTTCACACG |
| PSD2-F | GCCCTGTAGATGCCAAAGT |
| PSD2-R | GGGAGTGAAAGTGGTGGTAG |
